# Structural and functional perspectives on DEAD-box RNA helicases in the rubber tree cold stress response

**DOI:** 10.3389/fpls.2026.1959829

**Published:** 2026-09-15

**Authors:** Baocai Gao, Mingxia Xiao, Changli Mao, Jin Liu

**Affiliations:** Yunnan Technology Innovation Center of Natural Rubber, Yunnan Key Laboratory of Sustainable Utilization Research on Rubber Tree, Yunnan Institute of Tropical Crops, Jinghong, China

**Keywords:** AlphaFold2, cold stress, DExD/H-box family, RNA helicases, rubber tree, structural biology

## Abstract

DExD/H-box RNA helicases constitute one of the largest families of adenosine triphosphate (ATP)-dependent nucleic-acid enzymes, with roles spanning the full breadth of RNA metabolism, from transcription to turnover. Their defining architectural feature is a conserved helicase core comprising two tandem RecA-like domains that couples ATP hydrolysis to duplex unwinding and ribonucleoprotein remodeling. Structural investigations have historically focused on human and yeast model systems, but recent methodological advances, particularly cryo-electron microscopy and deep learning-based structure prediction, have broadened the structural coverage of the DDX family, including full-length constructs and their incorporation into multiprotein assemblies. In plants, DDX helicases remain far less characterized despite their essential functions in development and stress adaptation. This review integrates structural biology insights with recent multi-omics datasets from cold-stressed rubber tree (*Hevea brasiliensis*), a tropical crop highly susceptible to chilling injury. Quantitative profiling has identified nine differentially abundant DDX proteins under cold stress, among which DDX39B shows the most pronounced response. Rubber tree DDX39B shares >95% sequence identity with orthologs from other tropical species and ~73% identity with human DDX39B; AlphaFold2 predictions reveal near-identical core helicase folds, consistent with strong functional constraint across vast evolutionary distances. These predictions also reveal plant-specific domain arrangements, including a putative DDX39B isoform bearing additional LysM and protein kinase domains. This work illustrates how computational structure prediction can bridge the sequence-to-function gap for stress-responsive proteins in non-model plant species and provides a conceptual framework for dissecting DDX helicase roles in plant abiotic stress tolerance.

## 1 Introduction

RNA helicases form a vast superfamily of motor proteins that harness the chemical energy of ATP binding and hydrolysis to remodel RNA secondary structures, disassemble ribonucleoprotein (RNP) complexes, and drive conformational rearrangements of RNA molecules (Shi et al., 2004; Fairman-Williams et al., 2010; Su et al., 2021; Gao et al., 2022). Within this superfamily, the DEAD-box (DDX) family stands out: named for its signature Asp-Glu-Ala-Asp (DEAD) sequence in Walker B motif II, it is the largest and most thoroughly characterized group of RNA helicases (He et al., 2026; Hohmann et al., 2026). The architectural hallmark of DDX proteins is a conserved helicase core of two RecA-like domains (D1 and D2) that together contain at least twelve conserved sequence motifs involved in ATP coordination, RNA substrate recognition, and interdomain communication (Bai et al., 2021; Devasahayam Arokia Balaya et al., 2025; Bugai et al., 2026; Hia et al., 2026). Structural studies, from early X-ray crystallography to recent cryo-EM and AlphaFold2 predictions, have progressively unveiled the conformational dynamics and regulatory mechanisms of DDX helicases (Yin et al., 2015; Studer et al., 2020; Bugai et al., 2026).

Most DDX helicase research has focused on human and yeast model systems, but evidence indicates that DDX proteins play equally critical roles in plants, where they contribute to developmental patterning, hormone signaling, and responses to both abiotic and biotic stresses (Linder and Owttrim, 2009; Huang et al., 2016; Capel et al., 2020; He et al., 2021; Jia et al., 2025). In *Arabidopsis thaliana*, for example, the DEAD-box helicase *AtRH7*/*PRH75* participates in pre-rRNA processing and is required for cold tolerance (Huang et al., 2016), whereas DDX42 plays a critical role in pre-mRNA splicing under cold stress (Lu et al., 2020). More recently, an RNA helicase was shown to coordinate with iron signal regulators to alleviate chilling stress in *A. thaliana* (Xing et al., 2025). These studies establish that DEAD-box helicases are integral components of the plant cold-stress response network, yet their roles in tropical crop species remain largely unexplored.

Rubber tree (*Hevea brasiliensis*) is the world’s primary commercial source of natural rubber, a strategically vital industrial material with applications spanning the automotive, medical, and defense sectors (Tang et al., 2016; Liu et al., 2020; Chao et al., 2023; Niu et al., 2024). Native to the Amazon basin, rubber tree has evolved under warm and stable climatic conditions, rendering it highly susceptible to cold stress, a major environmental constraint that restricts its cultivation range and suppresses latex yield during cold spells (Sullivan et al., 2020; Gao et al., 2025; Wang et al., 2026). Commercially, rubber is cultivated primarily in Southeast Asia (Thailand, Indonesia, Vietnam, Malaysia), West Africa (Côte d’Ivoire, Nigeria, Liberia), and southern China (Yunnan, Hainan, Guangdong). In many of these regions, periodic cold fronts, particularly in the northern cultivation margins such as Yunnan Province in China and the highlands of mainland Southeast Asia, cause chilling injury, defoliation, and significant reductions in latex production (Tang et al., 2016; Liu et al., 2020). The economic impact is substantial: cold events can reduce annual rubber output by 10 to 30% in affected areas and limit the expansion of rubber cultivation into marginally suitable lands that could otherwise support smallholder livelihoods. Elucidating the molecular mechanisms underlying cold stress tolerance in the rubber tree is therefore of both fundamental biological significance and practical agricultural importance.

Recent multi-omics investigations have identified multiple DDX family proteins that are differentially abundant under cold stress in rubber tree, suggesting that DDX-mediated reprogramming of RNA metabolism constitutes an important component of the cold stress response (Mao et al., 2022; Gao et al., 2025). In this review, we synthesize current structural understanding of the DEAD-box helicase family, integrate recent proteomic evidence from cold-stressed rubber tree, and use AlphaFold2 structural prediction to examine the cold-responsive helicase DDX39B in detail. We further discuss the potential translational implications of these findings for rubber-tree improvement and cold-tolerance breeding.

## 2 Classification and structural framework of DEAD-box helicases

DEAD-box proteins belong to helicase superfamily 2 (SF2), one of the largest and most diverse helicase superfamilies (Fairman-Williams et al., 2010). Within SF2, the DExD/H-box helicases form a major subdivision that is further divided into two principal clades: the DEAD-box (DDX) family and the DEAH/RHA family. These clades are distinguished by the sequence of their Walker B motif II, DEAD (Asp-Glu-Ala-Asp) for DDX proteins and DEAH (Asp-Glu-Ala-His) for DEAH/RHA proteins, and by differences in their mechanistic properties and domain architectures (**Figure 1A**). Representative members of the DEAD-box family include DDX3X, DDX21, DDX39B/UAP56, eukaryotic initiation factor 4A (eIF4A), and DDX58/retinoic acid-inducible gene I (RIG-I), whereas DHX36, DHX9, and BRR2 exemplify the DEAH/RHA family. Throughout this review, we use “DEAD-box” or “DDX” to refer specifically to the DEAD-box clade and “DExD/H-box” when discussing the broader subdivision encompassing both clades.

**Figure 1:**
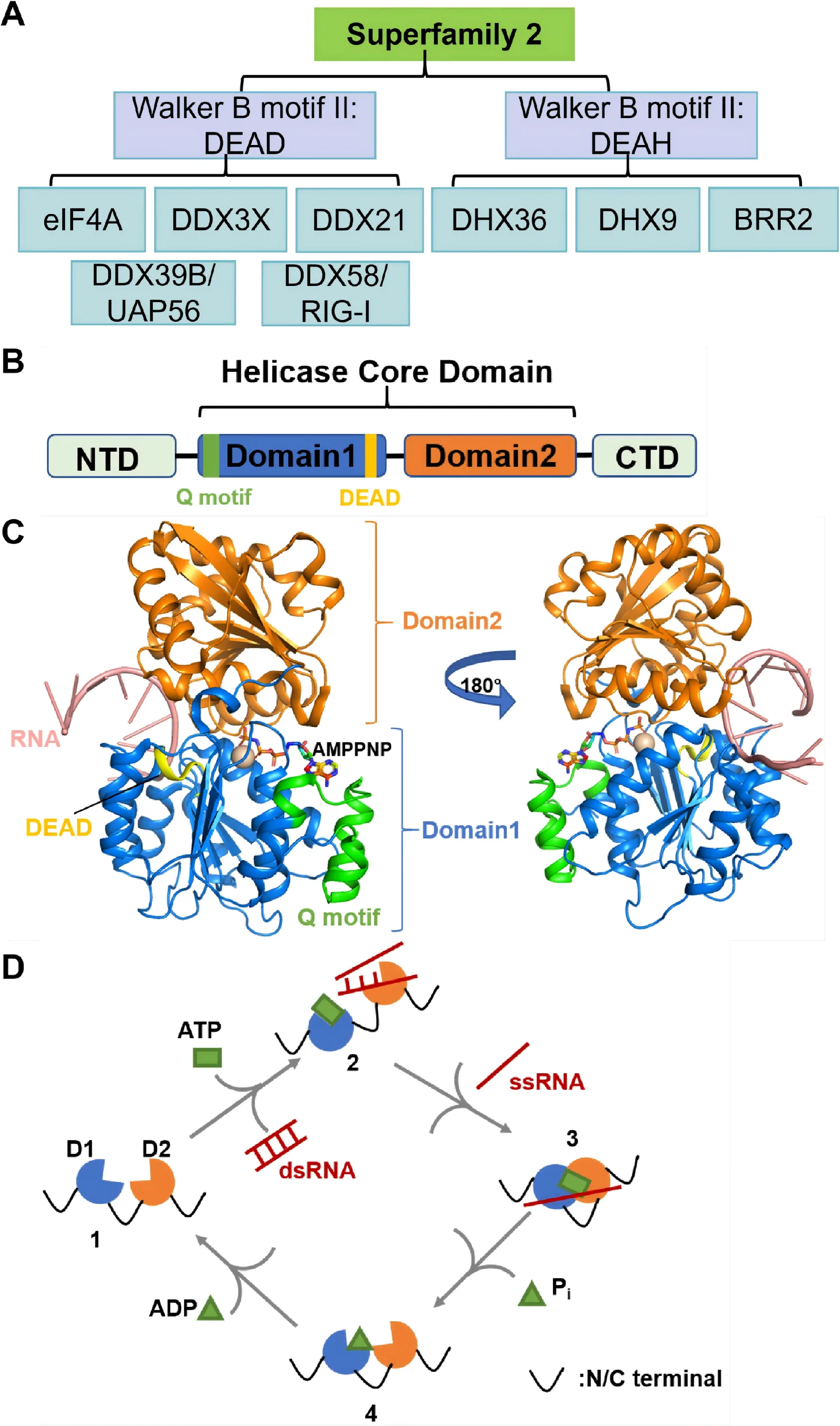
Classification and structural framework of DExD/H-box helicases. **(A)** Schematic classification of helicase superfamily 2 (SF2), illustrating the DExD/H-box subdivision and its two major clades: DEAD-box (DDX) and DEAH/RHA. Representative members for each clade are listed. **(B)** Domain architecture of the DEAD-box helicase core, composed of two tandem RecA-like domains (D1 and D2). The twelve conserved sequence motifs mediating ATP coordination, RNA-substrate recognition and inter-domain allosteric coupling are mapped onto the domains and color-coded according to their functional classes. **(C)** Crystal structure of the human DEAD-box helicase eIF4A1 core (PDB: 5ZC9), shown as a prototypical DEAD-box fold. Conserved helicase motifs are colored following the color scheme defined in **(B)**. **(D)** Schematic model for the DEAD-box ATPase cycle. In the nucleotide-free (apo) state, domains D1 and D2 adopt an open conformation with low intrinsic RNA-binding affinity. ATP binding within the inter-domain cleft triggers large-scale conformational rearrangement that closes the two domains, generating an extended RNA-binding surface. Subsequent ATP hydrolysis destabilizes the inter-domain interface, re-opening the cleft and driving the release of RNA product together with ADP and inorganic phosphate (Pi).

The defining architectural feature of DEAD-box helicases is a conserved catalytic core of two RecA-like domains (D1 and D2) that jointly coordinate ATP hydrolysis and RNA substrate recognition (**Figure 1B**). Twelve signature sequence motifs map to these two domains; some are shared broadly across SF2, whereas others, most notably the eponymous Asp-Glu-Ala-Asp (DEAD) sequence in motif II, are unique identifiers of the DEAD-box clade (**Figure 1C**). Variable N- and C-terminal extensions flank the core and underpin the family’s wide range of substrate specificities and biological functions (Cordin et al., 2006).

DEAD-box helicases operate via a cyclic series of conformational rearrangements. When unbound to RNA, the two RecA-like domains exhibit a range of open conformations and weak RNA-binding activity (**Figure 1D**). Engagement of dsRNA, coupled with ATP binding, drives closure of the interdomain cleft, generating a compact ternary complex that strains and unwinds the duplex. Hydrolysis of ATP then weakens the interdomain interface, promoting release of the unwound RNA strand and ADP-Pi products, and resetting the enzyme for subsequent catalytic rounds.

**Table 1** summarizes the structural data for DEAD-box family proteins curated from the Protein Data Bank in Europe (https://www.ebi.ac.uk/pdbe/). A striking pattern emerges from this data. Only a handful of full-length DDX proteins have been structurally characterized, including DDX2A and DDX8 (Napetschnig et al., 2009; Parsyan et al., 2011). The available structures, by contrast, represent truncated constructs encompassing primarily the helicase core. Many DDX structures have been determined in complex with binding partners, predominantly RNA-protein and protein-protein complexes. In recent years, cryo-electron microscopy has changed the landscape, facilitating structural analysis of an increasing number of full-length DDX family proteins involved in mRNA splicing, including DDX37, DDX38, and DDX46 (Yang et al., 2023; Liao et al., 2024; Hernández-Reyes et al., 2025).

**Table 1:** Reported DEAD-box family proteins with available structures.

Gene	Expression system	Sequence	Method	PDB ID	Ref.
DDX1	*E. coli*	72-283	X-ray	4XW3	2015 (Kellner and Meinhart, 2015)
DDX2A	*E. coli*	20-406	X-ray	2ZU6	2009 (Loh et al., 2009)
DDX2A	*E. coli*	1-406	cryo-EM	6ZMW	2020 (Brito Querido et al., 2020)
DDX2B	*E. coli*	22-240	X-ray	3BOR	2010 (Schütz et al., 2010)
DDX3X	*E. coli*	132-607	X-ray	6O5F	2019 (Song and Ji, 2019)
DDX4	*E. coli*	228-247	X-ray	5JIU	2016 (Hong et al., 2016)
DDX5	*E. coli*	52-304	X-ray	4A4D	2012 (Dutta et al., 2012)
DDX6	*E. coli*	95-469	X-ray	5ANR	2015 (Ozgur et al., 2015)
DDX6	*E. coli*	295-483	X-ray	6S8S	2019 (Peter et al., 2019)
DDX8	*HeLa*	1-1220	cryo-EM	6ICZ	2019 (Zhang et al., 2019)
DDX9	*E. coli*	1-91	X-ray	3VYY	2013 (Fu and Yuan, 2013)
DDX9	*E. coli*	152-264	X-ray	3VYX	2013 (Fu and Yuan, 2013)
DDX9	*E. coli*	329-563	X-ray	3LLM	2010 (Schütz et al., 2010)
DDX10	*E. coli*	47-280	X-ray	2PL3	2010 (Schütz et al., 2010)
DDX13	*Insect cells*	1-1246	cryo-EM	7QDR	2022 (Kögel et al., 2022)
DDX15	*Sf9 cells*	110-795	X-ray	5XDR	2017 (Murakami et al., 2017)
DDX15	*E. coli*	113-795	X-ray	6SHS	2020 (Studer et al., 2020)
DDX16	*Insect cells*	1-1041	cryo-EM	7DVQ	2021 (Bai et al., 2021)
DDX17	*E. coli*	32-477	X-ray	6UV3	2019 (Ngo et al., 2019)
DDX18	*E. coli*	149-387	X-ray	3LY5	2010 (Schütz et al., 2010)
DDX19B	*E. coli*	54-479	X-ray	6B4K	2018 (Lin et al., 2018)
DDX19B	*E. coli*	1-300	X-ray	3FMO	2009 (Napetschnig et al., 2009)
DDX20	*E. coli*	22-240	X-ray	3B7G	2010 (Schütz et al., 2010)
DDX21	*E. coli*	188-563	X-ray	6L5O	2020 (Chen et al., 2020)
DDX21	*E. coli*	617-710	NMR	2M3D	
DDX23	*E. coli*	338-820	X-ray	4NH0	2014 (Möhlmann et al., 2014)
DDX23	*Saccharomyces cerevisiae*	1-820	cryo-EM	6QW6	2019 (Charenton et al., 2019)
DDX25	*E. coli*	307-479	X-ray	2RB4	2010 (Schütz et al., 2010)
DDX26	*E. coli*	800-887	X-ray	7BV7	2021 (Jia et al., 2021)
DDX26	*HEK Expi293 cells*	1-887	cryo-EM	7CUN	2020 (Zheng et al., 2020)
DDX28	*HEK Flp-In 293 T-Rex cells*	1-540	cryo-EM	7OI6	2021 (Cheng et al., 2021)
DDX30	*E. coli*	42-147	NMR	2DB2	
DDX36	*E. coli*	53–70	NMR	2N16	2015 (Heddi et al., 2015)
DDX36	*E. coli*	53-81	X-ray	6Q6R	2020 (Heddi et al., 2020)
DDX37	*HEK Expi293 cells*	1-1157	cryo-EM	7MQA	2021 (Singh et al., 2021)
DDX38	*E. coli*	1-1227	cryo-EM	6ZYM	2020 (Bertram et al., 2020)
DDX39B	*E. coli*	1-428	cryo-EM	7APK	2020 (Pühringer et al., 2020)
DDX39B	*E. coli*	45-428	X-ray	1XTK	2004 (Shi et al., 2004)
DDX41	*E. coli*	153-410	X-ray	5H1Y	2017 (Jiang et al., 2017)
DDX41	*E. coli*	402-569	X-ray	2P6N	2010 (Schütz et al., 2010)
DDX46	*HeLa S3 cells*	1-1031	cryo-EM	6Y50	2020 (Zhang et al., 2020)
DDX47	*E. coli*	5-230	X-ray	3BER	2010 (Schütz et al., 2010)
DDX48	*E. coli*	1-411	X-ray	4C9B	2013 (Buchwald et al., 2013)
DDX50	Synthesis system	575-659	NMR	2E29	2009 (Ohnishi et al., 2009)
DDX52	*E. coli*	139-381	X-ray	3DKP	2010 (Schütz et al., 2010)
DDX53	*E. coli*	204-430	X-ray	3IUY	2010 (Schütz et al., 2010)
DDX58	*E. coli*	230-925	X-ray	2YKG	2011 (Luo et al., 2011)
DDX58	*E. coli*	1-201	X-ray	4P4H	2014 (Wu et al., 2014)
DDX59	Synthesis system	94-146	NMR	2YQP	

This table includes *Homo sapiens* proteins only. For the same protein with multiple structures, only representative data are shown. Data curated from the Protein Data Bank in Europe (https://www.ebi.ac.uk/pdbe/).

## 3 Structural features and functional diversity of representative DEAD-box helicases

In this section, we review the structural basis and functional diversity of representative DEAD-box and related DExD/H-box helicases. These examples, drawn primarily from human and animal systems where structural data are most abundant, illustrate the mechanistic paradigms that inform our understanding of plant homologs.

### 3.1 DDX3X: a prototypical DEAD-box helicase in RNA metabolism

DDX3X is a prototypical DEAD-box helicase that functions as a key regulator of RNA metabolism (Mo et al., 2021; Cai et al., 2025; Zhang et al., 2024). The human genome encodes two DDX3 paralogs, DDX3X and DDX3Y, sharing 92% sequence identity but differing markedly in expression: DDX3X is ubiquitously expressed across tissues, whereas DDX3Y is testis-restricted and required for spermatogenesis (You et al., 2023; Wang et al., 2025). DDX3X orthologs are deeply conserved across eukaryotes, consistent with their fundamental roles in RNA processing (Gadek et al., 2023; Ma et al., 2025). DDX3X participates in nearly every stage of the RNA life cycle, including transcription, pre-mRNA splicing, nuclear export, and translation (De Bortoli et al., 2021; Mo et al., 2021; Ullah et al., 2022; Weis and Hondele, 2022; Trussina et al., 2025).

The functional unit of DDX3X comprises the canonical D1D2 helicase core plus short N- and C-terminal extensions (NTE and CTE) required for full activity (**Figure 2A**). In the RNA-unbound state, structural analysis reveals that the two RecA-like domains no longer maintain a compact arrangement; instead, they attain an open V-shaped conformation, with their catalytic cores splayed apart and minimal interdomain contacts (Saito et al., 2024). Substrate binding drives a large conformational rearrangement that closes the interdomain cleft, generating a compact structure that encircles the RNA. The DDX3X D1D2 core bound to a 23-bp dsRNA (Protein Data Bank [PDB]: 6O5F), captured in an unwound state, reveals a 2:1 binding stoichiometry: two DDX molecules engage a single 2-trans A-form duplex, with each D1D2 unit primarily contacting one strand (Swarup and Bolger, 2024) (**Figure 2B**). The resulting D1D2:dsRNA:D1D2 assembly forms a molecular clip in which four RecA-like domains (two D1, two D2) sandwich the dsRNA. The NTE and CTE are proposed to facilitate ATP and RNA binding to DDX3X, respectively. Mechanistic studies reveal that the RNA-stimulated interaction between DDX3X and ATP requires an N-terminal ATP-binding loop (residues 152 to 160), while deletion of the RDYR motif within the CTE impairs RNA unwinding activity (Zhang et al., 2023; Tsai et al., 2024). Nuclear export signals (NES) identified in both terminal domains correlate with the tissue-specific subcellular localization of DDX3X, which localizes to either the cytoplasm or nucleus depending on cellular context (Swarup and Bolger, 2024). These findings demonstrate the functional importance of flanking regions in modulating DDX3X activity.

**Figure 2:**
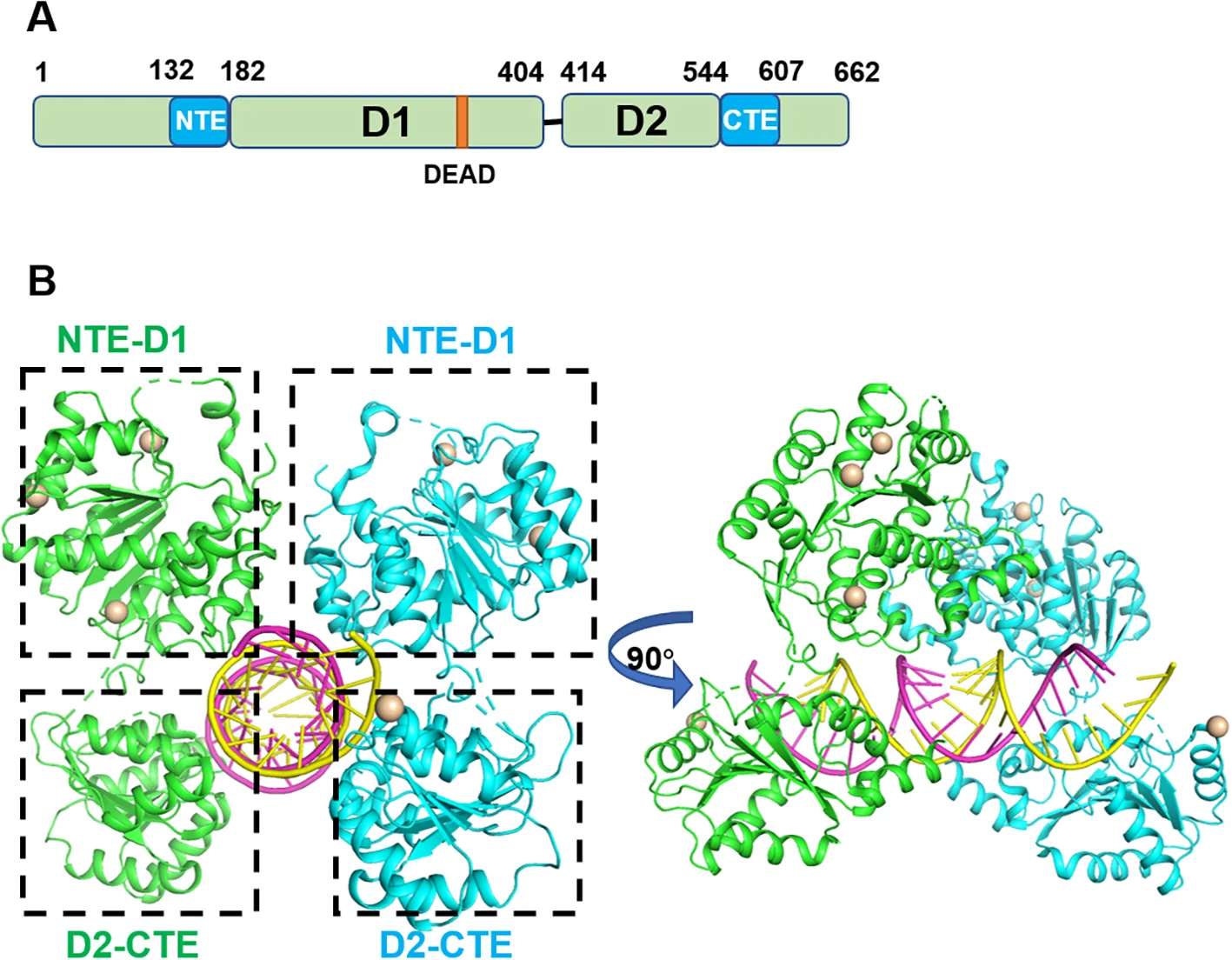
Overall structure of the D1D2:dsRNA:D1D2 assembly of human DDX3X. **(A)** Domain structure of human DDX3X and conserved sequence motifs of DDX-family helicases. The minimal active helicase-core construct (D1D2 core, residues 132-607) was used for structural determination. **(B)** Crystal structure of the D1D2:dsRNA:D1D2 assembly shown in two orthogonal views (PDB ID: 6O5F). The complex comprises two D1D2 polypeptide chains (green/cyan), one 23-bp double-stranded RNA (dsRNA) duplex (magenta/lemon), and seven chloride ions (wheat).

### 3.2 DDX21: nucleolar helicase in genome stability and R-loop resolution

DDX21 is a major nucleolar helicase whose role in genome stability has been implicated in R-loop-related stress (Chen et al., 2020; Wang et al., 2021; Chen et al., 2022; Wang et al., 2024). DDX21 depletion leads to RNaseH-dependent accumulation of DNA damage (Hao et al., 2024). Depletion of DDX21 leads to a concomitant elevation in DNA damage markers and DNA: RNA hybrid levels specifically at its chromatin-associated target genes, providing evidence that this helicase functions to prevent or dismantle R-loop structures under physiological conditions (Zhang et al., 2011; Lavergne and Roignant, 2024; Chen et al., 2026). R-loops play important physiological roles in transcription regulation, immunoglobulin class switching, and DNA replication (Lavergne and Roignant, 2024). Their unchecked accumulation can lead to replication stress, DNA damage, and genome instability (Tang et al., 2024).

DDX21 harbors signature motifs of the DExD-box family and exhibits RNA unwinding, ATPase, and G-quadruplex resolvase activities (Chen et al., 2020; Gao et al., 2023). Its helicase core adopts a canonical bilobed fold with two RecA-like domains (D1 and D2) connected by a flexible linker (**Figure 3A**). Crystal structures of the human DDX21 core (residues 188 to 563) have been captured in three distinct catalytic states: (i) AMPPNP-bound in complex with a 15-mer poly(U) ssRNA at 2.24 Å resolution (post-unwound/pre-hydrolysis) (**Figure 3B**), (ii) ADP-bound (post-hydrolysis/post-release) (**Figure 3C**), and (iii) apo state (no cofactor or RNA) (**Figure 3D**). Comparison of these structures reveals a canonical DEAD-box closure mechanism: substrate binding brings D1 and D2 into proximity, forming a composite ATPase active site and an RNA-binding cleft. ATP hydrolysis then drives the conformational cycle that underpins RNA unwinding and RNP complex disassembly (**Figure 3E**).

**Figure 3:**
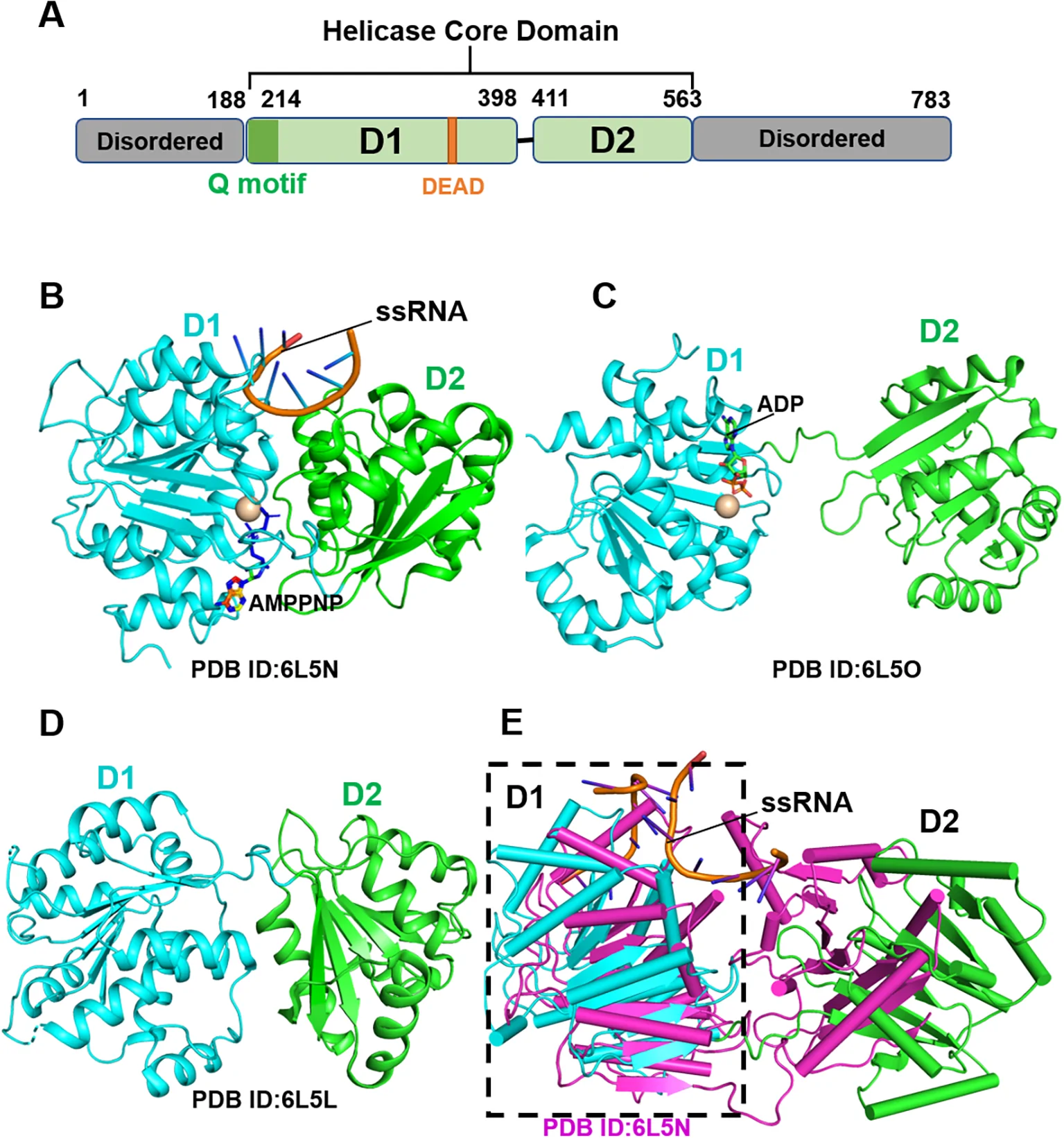
The overall structure of DDX21 helicase core in different unwinding states. **(A)** Domain architecture of human DDX21 and conserved sequence motifs within the helicase core. **(B)** Crystal structure of the D1D2 helicase core (yellow/red) in complex with ssRNA and AMPPNP-Mg²^+^. **(C)** Crystal structure of the D1D2 helicase core (cyan/green) bound to ADP-Mg²^+^. **(D)** Crystal structure of the D1D2 helicase core (cyan/green) in the apo-state. A magnesium ion is depicted as a wheat-colored sphere. **(E)** Conformational superposition between apo-DDX21 (D1: cyan; D2: green; PDB ID: 6L5L) and DDX21-ssRNA-AMPPNP (in magenta, PDB ID: 6L5N).

### 3.3 DHX36: G-quadruplex recognition and antiviral immunity

DHX36 is a member of the DEAH/RHA helicase family (a sister clade to DEAD-box within DExD/H-box) and binds DNA and RNA G-quadruplexes with high affinity (Jiao et al., 2025). DHX36 is a multifunctional helicase involved in G-quadruplex resolution, telomere maintenance, translation regulation, and antiviral innate immunity (Gómez-Del Arco et al., 2024; Zhang et al., 2024; Jiao et al., 2025; Sauer et al., 2019). Beyond immune sensing, DHX36 is a well-characterized RNA G-quadruplex (rG4) resolvase (Chang-Gu et al., 2021; Cheng et al., 2025). rG4s are non-canonical four-stranded nucleic acid structures found throughout the transcriptome that modulate gene expression by engaging G4-binding proteins (G4BPs) (Cheng et al., 2025; Sato et al., 2025).

DHX36 displays a modular domain organization typical of the DEAH/RHA helicase family. The N-terminal region contains a glycine-rich element and the DHX36-specific motif (DSM), which is critical for G-quadruplex recognition (**Figure 4A**). High-resolution structural data for full-length human DHX36 remain scarce; most available structures are of truncated constructs. The most detailed structural insights come from the co-crystal structure of bovine DHX36 bound to a DNA G-quadruplex bearing a 3′ single-stranded overhang. This structure reveals a trefoil architecture in which RecA1, RecA2, and the C-terminal domain adopt a three-lobed configuration (**Figure 4B**). The N-terminal extension, connected to RecA1 via a disordered linker, folds into two α-helices; the N-proximal helix harbors the DSM and extends outward from the helicase core, docking onto the 5′ (top) face of the G-quadruplex. The DSM α-helix and the OB subdomain confer selective recognition of parallel G-quadruplex topologies (Fu et al., 2024; Bartosik et al., 2025).

**Figure 4:**
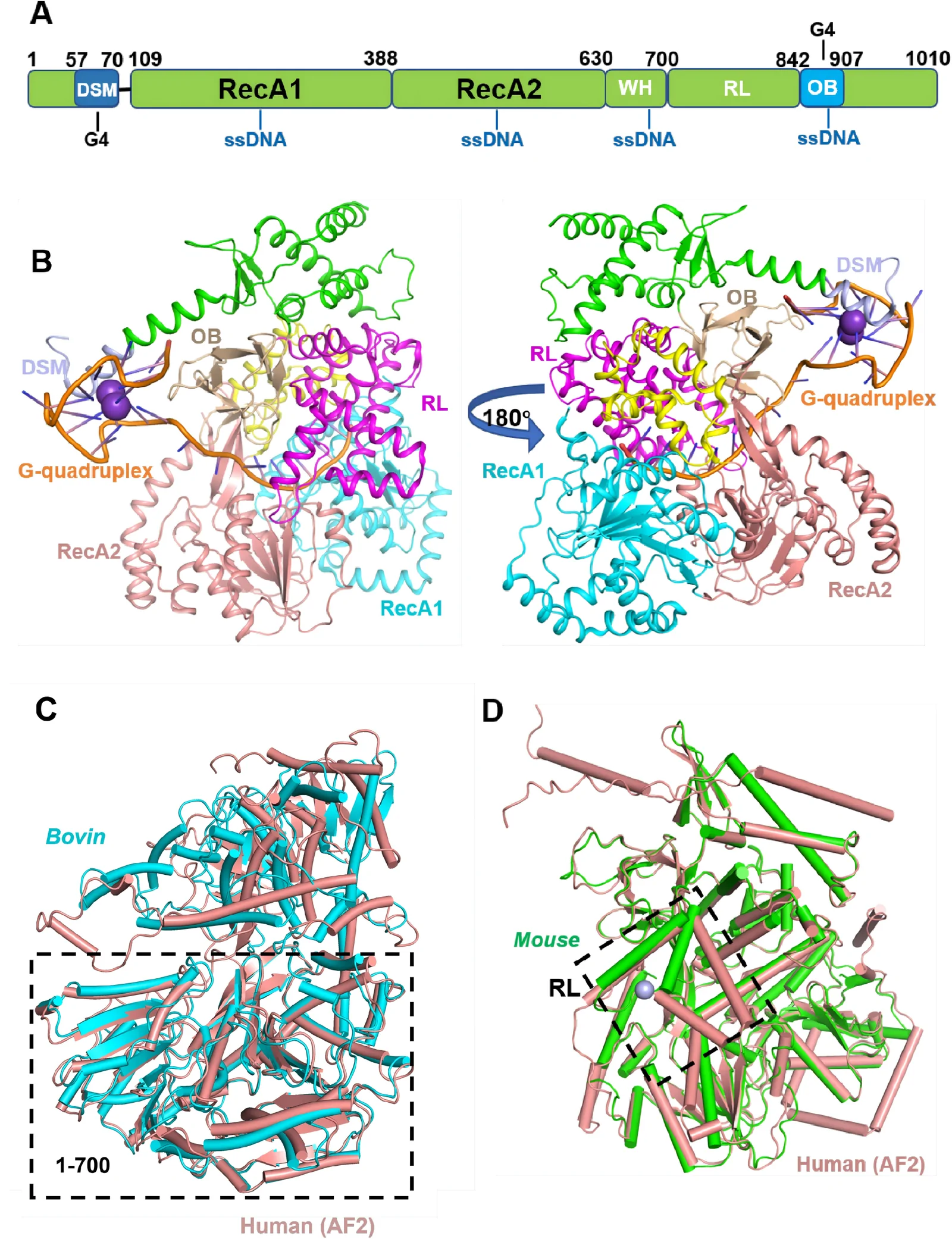
Overall structure of the DHX36-G-quadruplex DNA complex. **(A)** Domain architecture of DHX36; regions interacting with G-quadruplex (G4) and ssDNA are indicated. **(B)** Cartoon representation of the co-crystal structure of bovine DHX36 bound to DNA (PDB ID: 5VHE), color-coded as in **(A)** Spheres denote two disordered segments. OB loops contact DNA. DSM: DHX36-specific motif; RecA: RecA-like domains; WH: winged-helix; RL: ratchet-like; OB: oligonucleotide/oligosaccharide-binding fold. **(C)** Conformational superposition of human DHX36 (AF2) and bovine DHX36 (PDB ID: 5VHE). After exclusion of the low-confidence DSM region, RMSD = 1.876 Å (residues 1 to 700: RMSD = 1.509 Å). **(D)** Conformational superposition of human DHX36 (AF2) and mouse DHX36 (PDB ID: 6UP2), RMSD = 0.514 Å. AF2: AlphaFold2.

The structure of human DHX36 has not yet been experimentally determined. To address this gap, AlphaFold2 (AF2) was used to predict its structure, which was then compared with the crystal structures of bovine and mouse DHX36 (Tunyasuvunakool et al., 2021; Wayment-Steele et al., 2024). After removing the DSM region (low-confidence prediction of disordered linker regions), the RMSD between the human DHX36 AF2 model and bovine DHX36 crystal structure (PDB ID: 5VHE) was 1.876 Å; specifically, the RMSD for the amino acid 1 to 700 region was 1.509 Å, indicating that the core structural domains of the two proteins are highly conserved (**Figure 4C**). By contrast, the RMSD between the human DHX36 AF2 model and mouse DHX36 (PDB ID: 6UP2) was 0.514 Å, indicating higher structural similarity (**Figure 4D**). Multiple sequence alignment of DHX36 orthologs from human, bovine, and mouse confirms high sequence conservation across the core helicase domains, with divergence concentrated in the N-terminal glycine-rich and DSM regions (**Supplementary Figure S1**). These comparisons reveal that the human DHX36 model is less compact and contains more flexible regions than the bovine and mouse DHX36 crystal structures, potentially explaining the difficulty in crystallizing human DHX36.

### 3.4 RIG-I-like receptors: viral RNA sensing and conformational dynamics

RIG-I-like receptors, RIG-I (DDX58), MDA5, and LGP2, are cytoplasmic viral RNA sensors that trigger innate immunity and inflammation (Onomoto et al., 2021; Oshiumi et al., 2016; Ahmad et al., 2018; Thuner and Coutant, 2023; Chen et al., 2026). RIG-I and MDA5 have non-overlapping ligand specificities (Kato et al., 2021; Lang et al., 2017; Meng et al., 2017): RIG-I recognizes 5′-triphosphorylated ssRNA and blunt-ended dsRNA, whereas MDA5 detects long dsRNA duplexes independent of 5′ phosphorylation (Song et al., 2022; Pitré et al., 2025). Both receptors signal through the mitochondrial antiviral-signaling protein (MAVS), which activates IKK and TBK1 cascades to drive NF-κB activation and type I IFN production (Yin et al., 2015; Li et al., 2025; Nguyen et al., 2025; Kim et al., 2025).

RIG-I adopts a tripartite domain architecture: an N-terminal tandem caspase activation and recruitment domain (CARD) module, a central DExD/H-box helicase core, and a C-terminal regulatory domain (RD; also called repressor domain) (**Figure 5A**). The helicase core comprises two RecA-like domains, Hel1 and Hel2. In the autoinhibited apo state, Hel1 and Hel2 adopt an open, V-shaped arrangement; upon RNA engagement, the two domains undergo a large conformational rearrangement, closing into a compact ring that fully encircles the RNA ligand (**Figure 5B**). A notable structural feature is the pincer: a V-shaped element formed by two long α-helices that project from Hel2, connect to the C-terminal domain (CTD), and clasp an α-helix extending from Hel1. This pincer-CTD network creates a rigid structural framework that couples RNA binding to the allosteric activation of downstream signaling (Genoyer et al., 2025; Kirchhoff et al., 2025).

**Figure 5:**
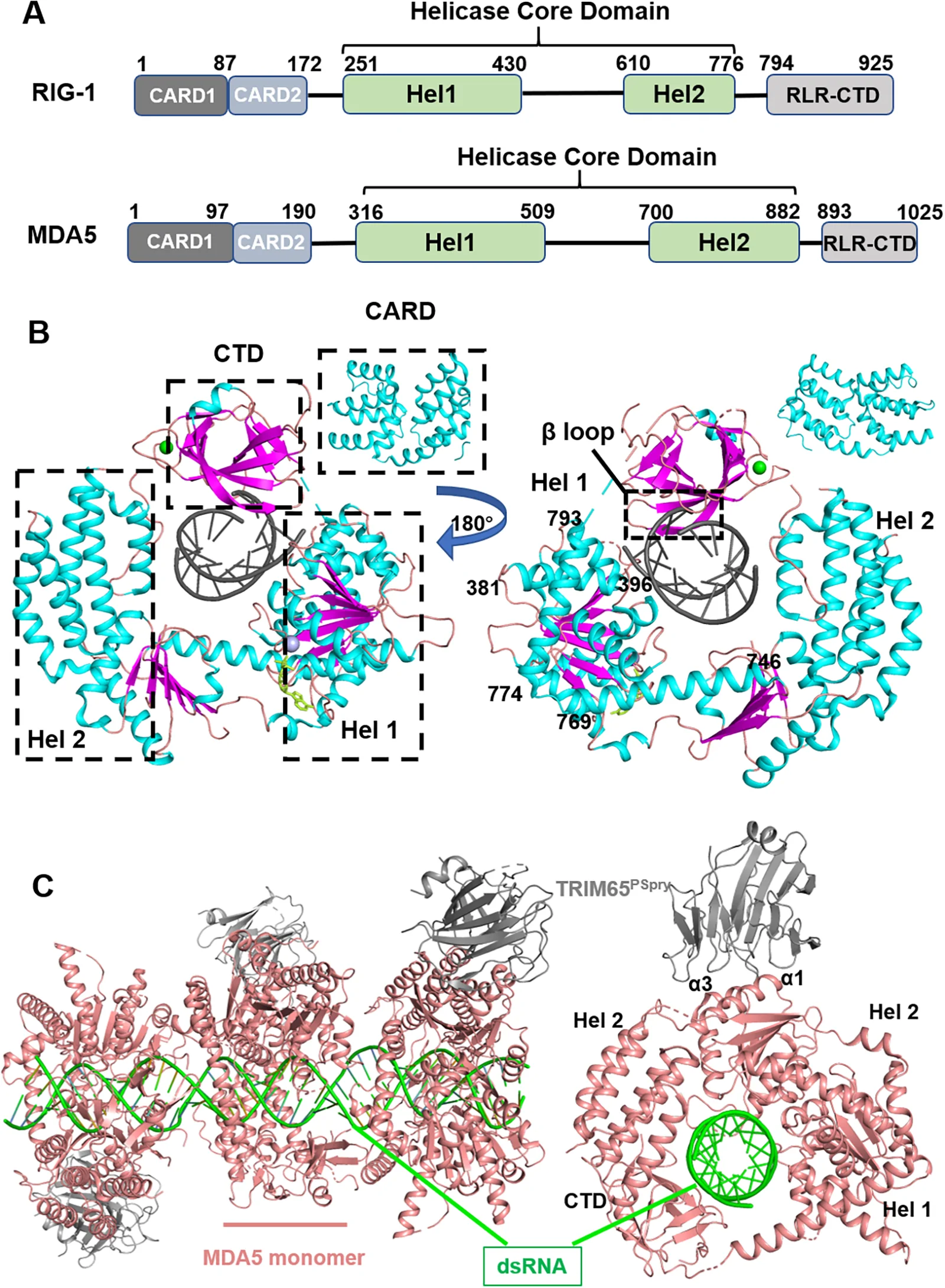
Interactions of RIG-I, MDA5, and dsRNA. **(A)** Domain architecture of RIG-I, featuring a C-terminal regulatory domain (CTD), a bipartite helicase core comprising two RecA-like subdomains (Hel1 and Hel2), and a pincer element bridging Hel2 and the CTD. **(B)** Crystal structure of human RIG-I (DDX58) bound to dsRNA (PDB ID: 3ZD7), viewed along the RNA axis. The helicase core together with the CTD forms a closed ring that fully encircles the dsRNA. Inset (top right): crystal structure of the tandem CARD signaling module in complex with MAVS-CARD (PDB ID: 4P4H). **(C)** Cryo-EM density map and ribbon model of the trimeric segment of the TRIM65PSpry-MDA5ΔN fusion filament (PDB ID: 7JL2). TRIM65PSpry, MDA5ΔN, and dsRNA are colored gray, salmon, and gray, respectively. Top view of the central monomeric complex of MDA5ΔN:TRIM65PSpry (PDB ID: 7JL0).

MDA5 shares the same domain organization as RIG-I but employs a fundamentally different activation mechanism: it assembles into helical filaments along the length of dsRNA (Kato et al., 2006; Lu et al., 2024). The E3 ubiquitin ligase TRIM65 regulates MDA5 activity through direct binding: its PRY/SPRY (PSpry) domain contacts the Hel2 subdomain of MDA5, engaging two adjacent α-helices (α1 and α3) on the outer surface of the filament (**Figure 5C**). Despite substantial conformational changes in the overall helicase core upon RNA and ATP binding, the Hel2 subdomain, particularly the α1/α3 region, maintains a stable conformation across monomeric and filamentous states (Dias Junior et al., 2019; Luo et al., 2026). These structural insights provide a foundation for understanding how RLR family helicases sense viral RNA and activate innate immune responses.

### 3.5 DEAD-box helicases in plant stress responses

While the structural paradigms above come primarily from animal systems, DEAD-box helicases are equally important in plants, where they have been implicated in development, hormone signaling, and abiotic stress responses (Linder and Owttrim, 2009; Li et al., 2023). Several plant DEAD-box helicases have been directly linked to cold or other abiotic stress tolerance. In *A. thaliana*, *AtRH7*/*PRH75*, a homolog of human DDX21, participates in pre-rRNA processing and is required for plant development and cold tolerance; *AtRH7* mutants show retarded growth and increased sensitivity to low temperatures (Huang et al., 2016). The DEAD-box helicase DDX42 plays a critical role in pre-mRNA splicing under cold stress, and its loss leads to defective splicing of cold-responsive genes and reduced chilling tolerance (Lu et al., 2020). More recently, an RNA helicase was shown to coordinate with iron signal regulators to alleviate chilling stress in *A. thaliana*, linking RNA metabolism to nutrient signaling under cold conditions (Xing et al., 2025). In tomato, the DEAD-box RNA helicase *SlDEAD39* has been implicated in plant development and salt response (Capel et al., 2020). These studies establish that DEAD-box helicases are integral components of the plant abiotic stress response network, and that the structural and mechanistic principles derived from animal homologs provide a valuable framework for interpreting their roles in non-model plant species such as rubber tree.

## 4 DDX helicases in rubber tree cold-stress response: from multi-omics data to structural prediction

As discussed in Section 1, rubber tree is a tropical crop highly susceptible to cold stress, and deciphering the molecular mechanisms underlying its cold stress response holds both fundamental and agricultural significance. Recent multi-omics investigations have revealed that RNA metabolism undergoes extensive reprogramming under cold stress conditions, with DEAD-box RNA helicases emerging as potentially important regulatory factors in this adaptive process (Mao et al., 2022; Gao et al., 2025). In this section, we summarize the identification and characterization of DDX-family proteins retrieved from rubber-tree cold-stress multi-omics datasets. We discuss their classification and putative functions derived from comparative sequence analysis and illustrate how AlphaFold2 (AF2) structural predictions can provide valuable mechanistic insights, building on the structural paradigms established in Section 3.

### 4.1 Identification of DDX family proteins in the rubber tree cold stress response

A previous quantitative analysis of cold-stressed rubber tree identified differentially abundant proteins in two cultivars: the cold-tolerant Yunyan77–4 and the cold-sensitive Reyan8-79 (Mao et al., 2022; Gao et al., 2025). Across ten comparison groups, nine differentially abundant DDX family proteins were identified (**Table 2**). RNA metabolic regulation thus operates at multiple levels, from transcription and splicing to translation and degradation, and forms an integral component of the rubber tree cold stress response network. These nine proteins fall into four functional categories: mRNA splicing/export (DDX39B isoforms), ribosome biogenesis (DDX47, DDX18, DDX21, DDX58), translation initiation (eIF4A), and RNA remodeling/quality control (DHX36, BRR2, HFM1) (Sloan and Bohnsack, 2018; Jagtap et al., 2023; Hausmann et al., 2024; Campbell et al., 2025).

**Table 2:** Cold stress-responsive RNA helicase proteins identified in rubber-tree.

Protein accession	Gene name	KEGG gene accession
A0A6A6KEA8	DDX39B/UAP56/SUB2	GH714_039376
A0A6A6LQY2	DDX39B/UAP56/SUB2	GH714_023848
A0A6A6M7Q9	DDX47/RRP3	GH714_001532
A0A6A6KHX2	DDX58	GH714_003141
A0A6A6MCP5	SNRNP200/BRR2	GH714_007547
A0A6A6N0L7	DDX18/HAS1	GH714_041308
A0A6A6LPP6	DHX36/RHAU	GH714_033802
A0A6A6M6P8	HFM1/MER3	GH714_010237
A0A6A6LHZ1	eIF4A	GH714_019340

Among the nine differentially abundant DDX proteins, DDX39B/UAP56 subfamily members are the most prominently cold-responsive, with two isoforms (A0A6A6KEA8 and A0A6A6LQY2) showing differential abundance across seven and six comparison groups, respectively. DDX39B mediates mRNA export and pre-mRNA splicing in yeast and animal systems (Pérez-Calero et al., 2020; Yellamaty and Sharma, 2024; Bugai et al., 2026; Hohmann et al., 2026), suggesting that stress-induced RNA metabolic reprogramming may be conserved across eukaryotes.

### 4.2 Structural characterization and evolutionary conservation of *Hb*DDX39B

Experimental structures of rubber tree DDX proteins are not yet available. However, AlphaFold2 (AF2) structure prediction offers a powerful computational alternative for inferring their structural features and functional mechanisms, the same approach used for human DHX36 in Section 3.3. *Hb*DDX39B localizes to the cytoplasm and displays a canonical DDX39B domain architecture: two tandem RecA-like helicase domains (D1 and D2), with no additional C-terminal extensions (Liu et al., 2020).

Multiple sequence alignment (**Supplementary Figure S2**) shows high evolutionary conservation of *Hb*DDX39B across divergent taxa (Gouet et al., 2003; Robert et al., 2025). *Hb*DDX39B (A0A6A6LQY2) shares >95% identity with orthologs from *Manihot esculenta* (A0A2C9UAV4), Theobroma cacao (A0AB32UNN5), and Durio zibethinus (A0A6P5ZQ42). This near-identity among tropical rosid species, whose last common ancestor lived tens of millions of years ago, is consistent with purifying selection.

To formally test purifying selection, we conducted a Ka/Ks (dN/dS, ω) analysis of DDX39B coding sequences from seven species: *Manihot esculenta*, *Hevea brasiliensis*, *Theobroma cacao*, *Durio zibethinus*, *Arabidopsis thaliana*, *Oryza sativa subsp. japonica*, and *Homo sapiens*. Protein sequences were aligned and back-translated to codon alignments; Ka and Ks were estimated by the Nei-Gojobori method with Jukes-Cantor correction (**Supplementary Figure S3**; **Supplementary Table S1**). All 14 informative pairwise comparisons returned ω far below unity, consistent with pervasive strong purifying selection. In dicots, mean ω = 0.017, with the minimum ω = 0.011 between *T. cacao* and *D. zibethinus* (99.3% protein-sequence identity). Monocot-dicot comparisons had moderately higher ω values (mean ω = 0.037), still indicative of strong selective constraint. Ancient-divergence pairs (plant-vertebrate, *Arabidopsis-rice*) exhibited Ks saturation that compromises ω inference, although ~71% protein-sequence identity reveals deep conservation. No comparison gave ω > 1. DDX39B is therefore under strong purifying selection across flowering plants.

Conservation extends to temperate plants (94% identity with *Arabidopsis thaliana*, A0A178UKF1) and even across the plant-animal divide (73% identity with human DDX39B/UAP56) (Mungall et al., 2003), reflecting deep evolutionary conservation of this essential mRNA metabolism factor since the last eukaryotic common ancestor.

### 4.3 Structural basis of cold-responsive regulation: DDX39B in RNA metabolic reprogramming

AlphaFold2 structural prediction of *Hb*DDX39B (A0A6A6LQY2) reveals a significant degree of structural conservation with its human ortholog (**Figure 6**, **Supplementary Figure S2**). The domain architecture is highly conserved between the two species (**Figure 6A**). Both have an N-terminal Q motif followed by two tandem RecA-like domains (D1 and D2) that form the canonical DEAD-box helicase core. The rubber tree ortholog spans 429 residues; the human ortholog, 428. Structural superposition of the AF2-predicted models shows near-perfect congruence between the two proteins (**Figure 6B**). The RMSD across the core helicase domain is only 0.299 Å, significantly low given that these proteins diverged over one billion years ago. Both D1 and D2 domains superimpose almost identically, as seen in the magnified views.

**Figure 6:**
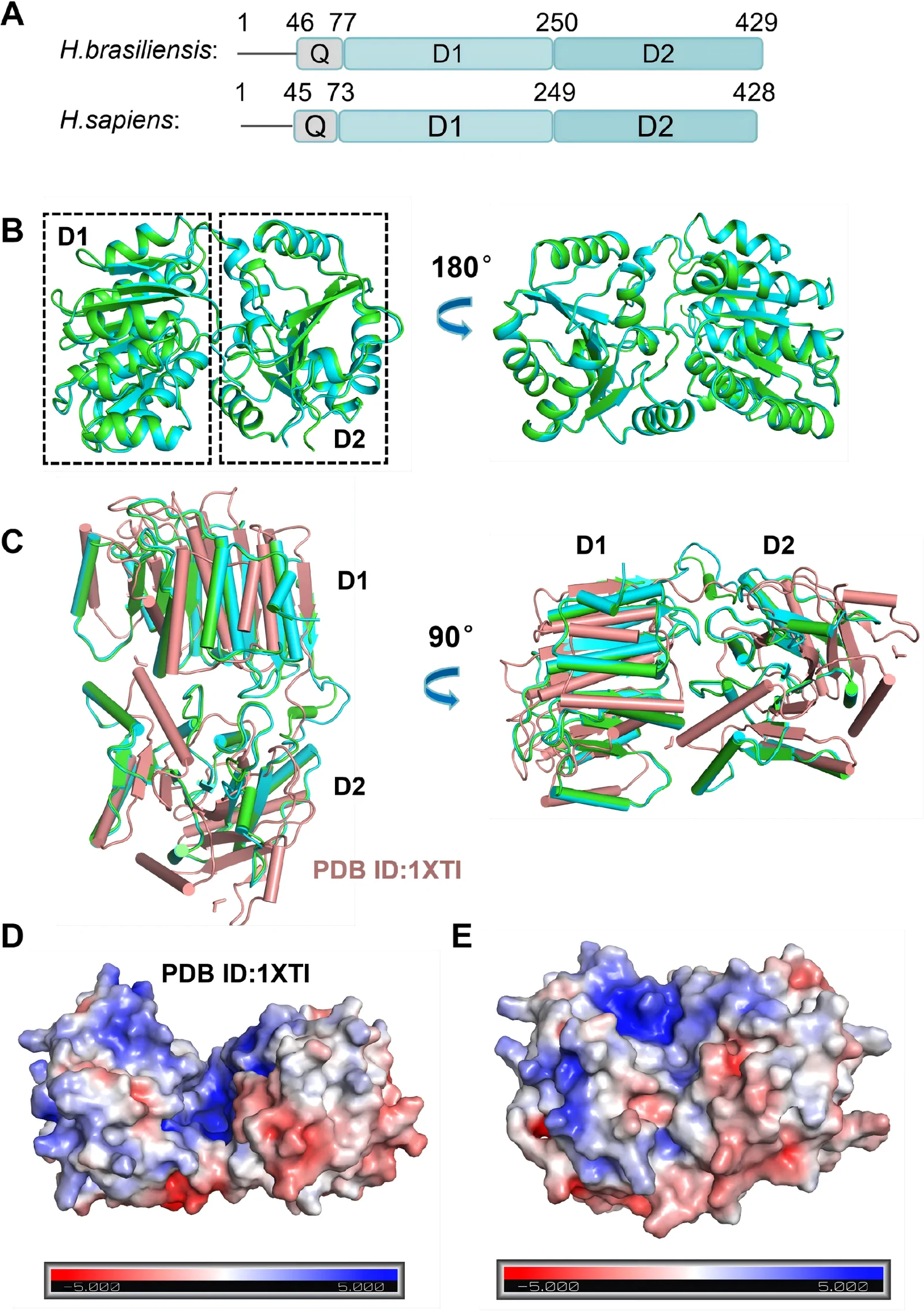
Comparative structural analysis of DDX39B from *H. sapiens* and *H. brasiliensis* based on AlphaFold2 predictions. **(A)** Domain architecture of human DDX39B and *H. brasiliensis* DDX39B. Q, Q motif; D1, helicase ATP-binding domain (RecA-like domain 1); D2, helicase C-terminal domain (RecA-like domain 2). **(B)** Structural superposition of AlphaFold2-predicted DDX39B models from the two species. Cyan, *H. brasiliensis* DDX39B; green, *H. sapiens* DDX39B. RMSD = 0.299 Å. **(C)** Structural superposition of three DDX39B structures. Cyan, *H. brasiliensis* DDX39B (AF2-predicted); green, *H. sapiens* DDX39B (AF2-predicted); salmon, crystal structure of human DDX39B (PDB ID: 1XTI). **(D)** Surface electrostatic potential map of the human DDX39B crystal structure (PDB ID: 1XTI). **(E)** Surface electrostatic potential map of the AlphaFold2-predicted *H. brasiliensis* DDX39B structure.

To further validate the AF2 predictions, both computational models were compared with the crystal structure of human DDX39B (PDB ID: 1XTI), an experimentally determined structure (Shi et al., 2004). All three structures superimpose well, supporting that the AF2-derived model of rubber-tree DDX39B adopts the canonical DEAD-box helicase core fold (**Figure 6C**). The surface electrostatic potential distributions are also similar between the human crystal structure and the rubber tree predicted model (**Figure 6D, E**). Both show a characteristic pattern: positively charged regions flanking the RNA-binding cleft and negatively charged patches on the opposite face. The near-identity in fold, surface charge, and domain organization across such vast evolutionary distance is consistent with strong functional constraint on DDX39B in core RNA metabolic processes. We caution, however, that these are computational structural predictions, and that higher-precision structures remain to be resolved through experimental approaches such as X-ray crystallography or cryo-EM.

The high conservation of DDX39B among tropical plants, coupled with its pronounced down-regulation under cold stress, carries important implications for understanding the molecular basis of tropical plant cold sensitivity. In contrast to temperate plants, which have evolved cold acclimation mechanisms involving C-repeat binding factor/dehydration-responsive element-binding (CBF/DREB) transcription factors and extensive transcriptomic reprogramming, tropical plants generally lack the capacity for cold acclimation and suffer severe damage even at moderately low temperatures (Taylor et al., 2019; Crous et al., 2022; Zhu et al., 2024; Liu et al., 2026). The observation that DDX39B is highly conserved in tropical plants and is strongly down-regulated by cold suggests that the cold sensitivity of tropical plants may be partially attributable to the inherent vulnerability of their RNA metabolic machinery to low temperatures. The high sequence conservation likely reflects a historical absence of selective pressure for cold-adaptive mutations in tropical environments, resulting in a DDX39B enzyme that is optimized for warm temperatures but may lose catalytic efficiency or become misregulated under cold conditions.

From a mechanistic perspective, the cold-induced down-regulation of DDX39B could impact several downstream RNA metabolic processes. As a homolog of yeast Sub2 and human UAP56, DDX39B is known to function in pre-mRNA splicing and mRNA nuclear export (Pérez-Calero et al., 2020; Hohmann et al., 2026). Under cold stress, reduced DDX39B abundance could lead to impaired splicing and nuclear export of cold-responsive transcripts, including those encoding CBF/DREB transcription factors, cold-regulated (COR) proteins, and enzymes involved in cryoprotective metabolite synthesis, thereby attenuating the cold acclimation response. Alternatively, DDX39B down-regulation may represent an adaptive energy-conservation mechanism, slowing energy-intensive RNA processing under stress conditions. Distinguishing between these possibilities will require targeted functional studies, including transcriptome-wide analysis of splicing and mRNA export changes in DDX39B-manipulated rubber tree lines under cold stress. The identification of specific RNA substrates of DDX39B in rubber tree, through techniques such as CLIP-seq or RIP-seq, would provide critical mechanistic links between cold stress, DDX39B activity, and downstream physiological responses.

## 5 Concluding remarks and future perspectives

This review synthesizes current structural knowledge of the DExD/H-box helicase family and integrates recent multi-omics evidence from rubber tree. It identifies DDX39B as a potentially important component of the cold-responsive RNA metabolic network, whose deep evolutionary conservation points to a fundamental role in RNA metabolic homeostasis. The integrative framework adopted here, combining multi-omics datasets with AlphaFold2 structure prediction, provides a generalizable strategy for mechanistic investigations in non-model plant species. The identification of DDX39B as a candidate cold-responsive factor in rubber tree, coupled with its high sequence conservation across tropical plant species, highlights several key priorities for future experimental work.

### 5.1 Structural validation and temperature-dependent enzymology

Computational structure prediction, while powerful, cannot substitute for experimentally determined structures. High-resolution crystal or cryo-EM structures of *Hb*DDX39B in its apo state and in complex with RNA substrates and non-hydrolyzable ATP analogs would validate the AF2 model and enable precise mapping of catalytic residues and RNA-binding interfaces. Comparative structural analysis with DDX39B orthologs from cold-tolerant temperate species (*e.g.*, *Arabidopsis thaliana*, *Brassica rapa*) may reveal subtle but functionally significant differences in domain packing, hydrogen-bond networks, or surface charge distribution that modulate helicase activity at low temperatures (Huang et al., 2016; Lu et al., 2020; Pandey et al., 2020; Li et al., 2023; Xing et al., 2025). Such comparisons could provide a structural basis for the observation that tropical plants are markedly more sensitive to chilling injury than their temperate counterparts, a phenomenon whose molecular determinants remain poorly defined (Li et al., 2024; Lai et al., 2025; Zhou et al., 2025).

### 5.2 Functional dissection of DDX39B in cold tolerance

A central unresolved question is whether DDX39B downregulation represents an adaptive energy-conservation mechanism or a passive consequence of cold-induced cellular damage that contributes to chilling injury. Distinguishing between these possibilities is critical for understanding the role of RNA metabolic reprogramming in the cold stress response. The AF2 structural model provides a rational framework for designing separation-of-function mutations that selectively disrupt ATPase activity, RNA binding, or protein-protein interactions, enabling precise dissection of which biochemical activities underlie the cold response phenotype. Identifying the upstream signaling pathways that modulate DDX39B expression, whether through cold-responsive transcription factors (e.g., the CBF/DREB regulon) or post-translational modifications (e.g., phosphorylation, SUMOylation), will clarify how cold signals are transduced to the RNA metabolism machinery. Transcriptome-wide splicing analysis and mRNA export assays in DDX39B-manipulated lines would further establish the mechanistic links between DDX39B activity and cold-responsive gene expression.

### 5.3 The A0A6A6KEA8 isoform: a putative chimeric helicase

The predicted LysM and protein kinase domains in the nuclear DDX39B isoform A0A6A6KEA8 represent a potentially novel domain architecture not previously reported in the DDX39B subfamily (Gust et al., 2012). If validated experimentally, this isoform could constitute a new class of multifunctional helicases that integrate RNA metabolic functions with stress signaling, perhaps by sensing cell wall-derived signals through the LysM domain or modulating pathway activity via kinase-mediated phosphorylation (Buendia et al., 2018; Tan et al., 2025). Systematic characterization of this isoform, including domain deletion analysis, subcellular localization studies, and interaction proteomics, promises to uncover previously unrecognized modes of helicase regulation in plants.

### 5.4 Translational implications for tropical crop improvement

The findings presented here have potential translational implications for rubber tree improvement and, more broadly, for tropical crop cold tolerance. Rubber cultivation is currently limited to tropical and subtropical regions where temperatures remain consistently warm; in marginal cultivation areas, such as the highlands of mainland Southeast Asia, parts of southern China (Yunnan, Guangxi), and emerging cultivation zones in West Africa, periodic cold events cause significant yield losses and restrict the geographic expansion of rubber farming. The development of cold-tolerant rubber varieties could therefore expand cultivation into marginally suitable lands, enhance production stability in existing regions, and improve the resilience of smallholder livelihoods dependent on rubber.

The >95% sequence identity of DDX39B among tropical species, including cassava (*Manihot esculenta*), cacao (*Theobroma cacao*), and jatropha (*Jatropha curcas*), all of which are economically important and cold-sensitive, suggests that findings from rubber trees may have broad translational relevance. If DDX39B-mediated RNA metabolic reprogramming is indeed a conserved determinant of tropical plant cold sensitivity, targeted modulation of its expression or activity could provide a general strategy for engineering chilling tolerance across multiple crop species. The *Hb*DDX39B structural model reported here provides a foundation for structure-guided protein engineering aimed at enhancing helicase stability or catalytic efficiency at low temperatures. DDX39B itself represents a promising target for molecular breeding, either through marker-assisted selection of favorable alleles or via transgenic approaches, to develop cold-tolerant rubber tree varieties suitable for cultivation in marginally suitable regions (Wei et al., 2025). A conceptual framework linking DDX39B-mediated cold stress responses to potential agricultural applications is presented in **Figure 7**.

**Figure 7:**
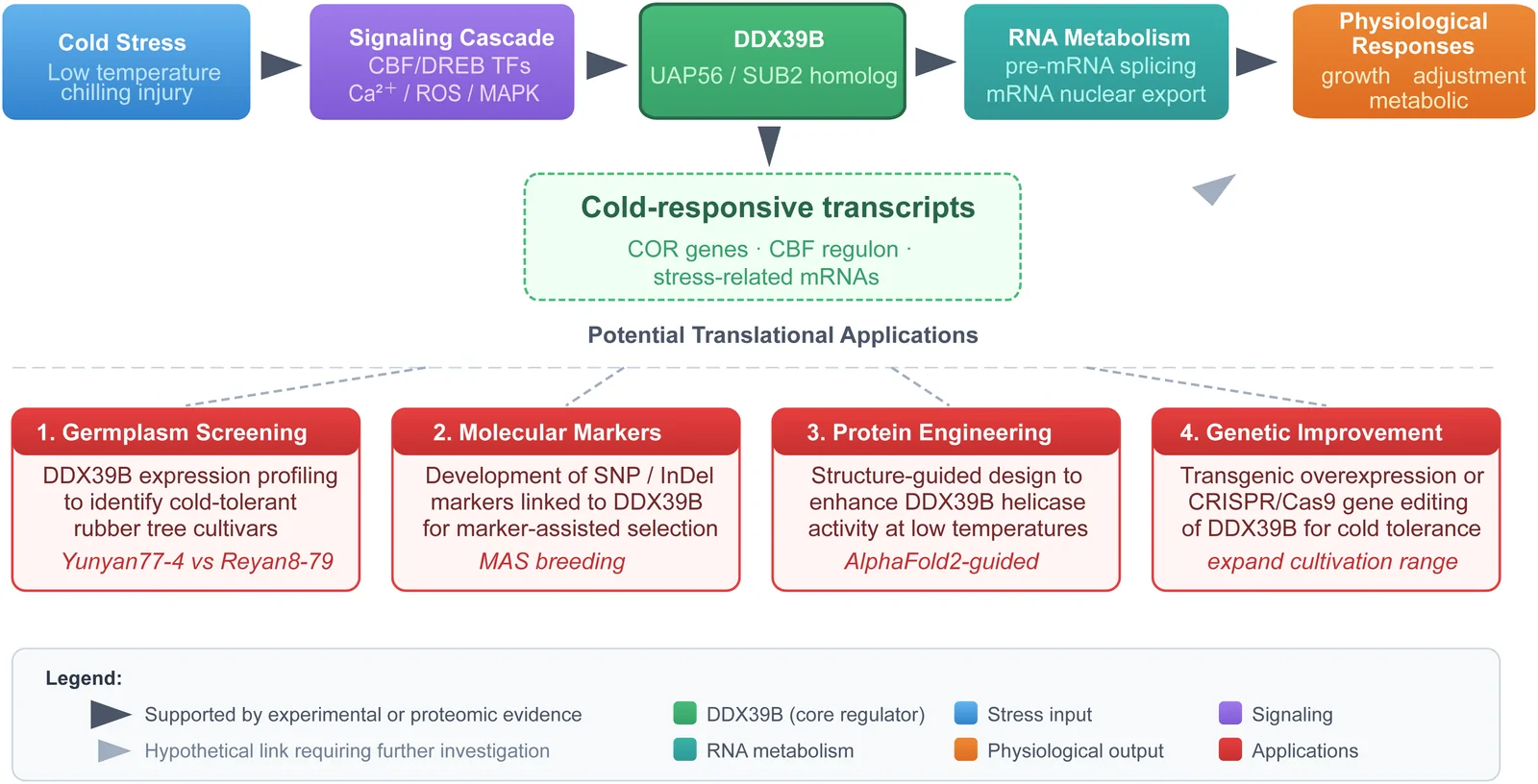
Conceptual model linking DDX39B-mediated cold stress responses to potential applications in rubber-tree improvement. Schematic model illustrating the proposed role of DDX39B in the rubber-tree cold-stress response and its potential translational applications. Cold stress is perceived by yet-uncharacterized sensors, which trigger signaling cascades (potentially involving CBF/DREB transcription factors) that modulate DDX39B expression and/or activity. In turn, DDX39B regulates pre-mRNA splicing and mRNA nuclear export of cold-responsive transcripts, and thereby shapes downstream physiological outcomes, including growth adjustment, metabolic reprogramming and cold-tolerance phenotypes. Solid arrows denote mechanistic links supported by experimental or proteomic evidence; dashed arrows represent hypothetical relationships requiring further experimental validation. Potential applications are outlined as follows: (1) identification of cold-tolerant germplasm via DDX39B expression profiling; (2) development of molecular markers for marker-assisted selection; (3) structure-guided protein engineering to enhance DDX39B activity under low-temperature conditions; and (4) transgenic or gene-editing strategies to generate cold-tolerant rubber-tree varieties suitable for cultivation in marginally suitable planting regions.

### 5.5 Methodological outlook

The integrative strategy presented here, combining quantitative multi-omics, AlphaFold2 structure prediction and cross-species comparative analysis, constitutes a generalizable conceptual framework to facilitate functional characterization of stress-responsive proteins in non-model organisms. As structural-prediction tools advance and multi-omics datasets for diverse plant species continue to expand, this computational approach holds growing potential to bridge the gap between large-scale omics discoveries and mechanistic interpretations in plant-stress biology.
