# Supplementary material for: Structural and functional perspectives on DEAD-box RNA helicases in the rubber tree cold stress response
Source: Front Plant Sci. 2026 Sep 15;17:1959829. doi: 10.3389/fpls.2026.1959829 (PMC13621893; doi:10.3389/fpls.2026.1959829)
Supplement: Supplementary file 1 [file DataSheet1.zip › Supplementary material/Supplementary material.docx]

**Supplementary material**

**Supplementary Figure S1. Multiple sequence alignment of DHX36 orthologs from *Homo sapiens* (human), *Bos taurus* (bovine), and *Mus musculus* (mouse).**

Residues identical across all three sequences are shaded red with white lettering; conserved physicochemical substitutions are shown in red lettering on a white background. The alignment spans the full-length protein (~1000 residues) and highlights strong conservation within the core helicase domains (RecA1, RecA2, WH, OB), with divergence concentrated in the N-terminal glycine-rich and DSM regions. Alignment generated with ClustalX and rendered using ESPript v3.0.

**Supplementary Figure S2. Multiple amino acid sequence alignment of DDX39B orthologs from different species.**

Multiple sequence alignment of DDX39B orthologs from representative species was generated with ClustalX and rendered using ESPript v3.0. Residues identical across all sequences are shaded red with white lettering; residues with conserved physicochemical properties are shown in red lettering on a white background. The alignment spans the full helicase core-encompassing both RecA-like domains (D1 and D2).

**Supplementary Figure S3. Pairwise Ka, Ks, and Ka/Ks (ω) heatmaps for DDX39B orthologs across seven species.**

Heatmaps showing (A) nonsynonymous substitution rate (Ka), (B) synonymous substitution rate (Ks), and (C) the Ka/Ks ratio (ω) for all pairwise comparisons of DDX39B coding sequences from *Manihot esculenta* (cassava), *Hevea brasiliensis* (rubber tree), *Theobroma cacao* (cacao), *Durio zibethinus* (durian), *Arabidopsis thaliana*, *Oryza sativa subsp. japonica* (rice), and *Homo sapiens* (human). Protein sequences were aligned by global pairwise alignment and back-translated to codon alignments; Ka and Ks were estimated using the Nei-Gojobori method with Jukes-Cantor correction. Diagonal cells (-) denote self-comparisons. "sat" indicates Ks saturation (pS ≥ 0.75) due to ancient divergence, precluding reliable ω estimation. All informative ω values are far below 1 (range 0.011-0.044), indicating strong purifying selection. Color scales are independent for each panel; note that panel (C) is capped at ω = 0.05 to visualize the narrow range of purifying selection ratios.

**Supplementary Table S1**

*Pairwise Ka/Ks (dN/dS) analysis of DDX39B homologous coding sequences across seven species*

| **No.** | ***Species pair*** | **Ka** | **Ks** | **Ka/Ks (ω)** | **S sites** | **N sites** | **Sd** | **Nd** | **Comparison type** |
| --- | --- | --- | --- | --- | --- | --- | --- | --- | --- |
| 1 | *T. cacao vs D. zibethinus* | 0.0030 | 0.2684 | 0.0111 | 275 | 1009 | 62.0 | 3.0 | Dicot-Dicot |
| 2 | *M. esculenta vs D. zibethinus* | 0.0125 | 1.0356 | 0.0121 | 275 | 1006 | 154.5 | 12.5 | Dicot-Dicot |
| 3 | *M. esculenta vs A. thaliana* | 0.0315 | 2.3381 | 0.0135 | 275 | 1006 | 197.0 | 31.0 | Dicot-Dicot |
| 4 | *T. cacao vs A. thaliana* | 0.0321 | 2.2086 | 0.0145 | 274 | 1007 | 194.3 | 31.7 | Dicot-Dicot |
| 5 | *M. esculenta vs T. cacao* | 0.0125 | 0.8538 | 0.0147 | 274 | 1007 | 139.5 | 12.5 | Dicot-Dicot |
| 6 | *H. brasiliensis vs A. thaliana* | 0.0362 | 2.1267 | 0.0170 | 276 | 1005 | 194.5 | 35.5 | Dicot-Dicot |
| 7 | *D. zibethinus vs A. thaliana* | 0.0316 | 1.8537 | 0.0171 | 275 | 1006 | 188.8 | 31.2 | Dicot-Dicot |
| 8 | *H. brasiliensis vs D. zibethinus* | 0.0176 | 0.8526 | 0.0207 | 276 | 1005 | 140.5 | 17.5 | Dicot-Dicot |
| 9 | *H. brasiliensis vs T. cacao* | 0.0176 | 0.7061 | 0.0249 | 274 | 1007 | 125.5 | 17.5 | Dicot-Dicot |
| 10 | *M. esculenta vs H. brasiliensis* | 0.0065 | 0.2394 | 0.0271 | 276 | 1005 | 56.5 | 6.5 | Dicot-Dicot |
| 11 | *D. zibethinus vs O. sativa* | 0.0664 | 2.1469 | 0.0309 | 277 | 1007 | 196.0 | 64.0 | Monocot-Dicot |
| 12 | *T. cacao vs O. sativa* | 0.0654 | 1.9649 | 0.0333 | 275 | 1006 | 191.0 | 63.0 | Monocot-Dicot |
| 13 | *M. esculenta vs O. sativa* | 0.0651 | 1.6589 | 0.0392 | 276 | 1005 | 184.3 | 62.7 | Monocot-Dicot |
| 14 | *H. brasiliensis vs O. sativa* | 0.0700 | 1.5973 | 0.0439 | 277 | 1004 | 182.8 | 67.2 | Monocot-Dicot |
| 15 | *A. thaliana vs O. sativa* | 0.0644 | saturated | NA | 276 | 1005 | 213.0 | 62.0 | Monocot-Dicot |
| 16 | *H. sapiens vs O. sativa* | 0.2082 | saturated | NA | 279 | 996 | 241.8 | 181.2 | Plant-Animal |
| 17 | *H. brasiliensis vs H. sapiens* | 0.2147 | saturated | NA | 274 | 986 | 234.8 | 184.2 | Plant-Animal |
| 18 | *M. esculenta vs H. sapiens* | 0.2174 | saturated | NA | 276 | 993 | 240.5 | 187.5 | Plant-Animal |
| 19 | *D. zibethinus vs H. sapiens* | 0.2181 | saturated | NA | 276 | 996 | 230.5 | 188.5 | Plant-Animal |
| 20 | *A. thaliana vs H. sapiens* | 0.2185 | saturated | NA | 274 | 986 | 236.2 | 186.8 | Plant-Animal |
| 21 | *T. cacao vs H. sapiens* | 0.2197 | saturated | NA | 275 | 997 | 229.0 | 190.0 | Plant-Animal |

**Summary statistics**

| **Comparison type** | **n** | **Mean Ka** | **Mean Ks** | **Mean ω** | **ω range** |
| --- | --- | --- | --- | --- | --- |
| Dicot–Dicot | 10 | 0.0201 | 1.2483 | 0.0173 | 0.0111–0.0271 |
| Monocot–Dicot | 4 | 0.0667 | 1.8420 | 0.0368 | 0.0309–0.0439 |
| Plant–Animal | 6 | 0.2161 | saturated | NA | — |
| All informative pairs | 14 | 0.0334 | 1.4179 | 0.0229 | 0.0111–0.0439 |

**Notes:**Ka, number of nonsynonymous substitutions per nonsynonymous site; Ks, number of synonymous substitutions per synonymous site; ω = Ka/Ks ratio.S sites, number of synonymous sites (averaged between the two sequences); N sites, number of nonsynonymous sites; Sd, number of synonymous differences; Nd, number of nonsynonymous differences.Ka and Ks were estimated using the Nei-Gojobori (NG) method with Jukes-Cantor (JC) correction for multiple substitutions. Protein sequences were aligned by global pairwise alignment (Needleman–Wunsch algorithm) and back-translated to codon alignments before Ka/Ks calculation. "saturated" indicates Ks exceeds the reliable detection limit (pS ≥ 0.75) due to ancient divergence, making Ka/Ks estimation unreliable (NA). All informative pairwise comparisons show ω << 1 (range 0.011–0.044; mean 0.023), indicating strong purifying selection. No comparison shows ω > 1 (positive selection). Species: *M. esculenta* (cassava), *H. brasiliensis* (rubber tree), *T. cacao* (cacao), *D. zibethinus* (durian), *A. thaliana* (thale cress), *O. sativa* (rice), *H. sapiens* (human).
